# The main factors affecting Taiwan’s economic growth rate via dynamic grey relational analysis

**DOI:** 10.1371/journal.pone.0240065

**Published:** 2020-10-05

**Authors:** Chiung-Yu Huang, Chia-Chin Hsu, Mu-Lin Chiou, Chun-I Chen

**Affiliations:** 1 Department of Nursing, I-Shou University, Kaohsiung, Taiwan; 2 Department of Industrial Management, I-Shou University, Kaohsiung, Taiwan; University of Defence, SERBIA

## Abstract

Ever since the grey system theory was proposed about 40 years ago, its characteristics such as small samples, few data, and uncertainty have been used for study in the literature with increasingly wider scope. Recent studies on grey relation analysis have included static data analyses, and most of them have adopted initial values with only a relational order. Under the same study conditions, if different data preprocessing methods are used, then the relational order will be ranked differently. This study took Taiwan as the object to explore seven economic indices (birth rate (%), Taiwan’s total population (thousand people), unemployment rate (%), income per capita (USD), weighted average interest rate on deposits (%), Consumer Price Index (CPI), and national income (NI)) and how they affect the economic growth rate. The traditional static grey relational analysis treated the collected data with taking consideration of time effect which is irrational under some circumstance. An innovative dynamic grey relational analysis was carried out by shifting the raw data due to the time leading or lagging effect which is a mean to improve the capability of traditional grey relational analysis. The differences in analyses between static grey relational analysis and dynamic grey relational analysis via different data preprocessing methods were further discussed, finding that different data preprocessing methods generated a new set of relational orders through the latter. Finally, the prosperity index was used to identify the effects of all factors on economic growth (leading, synchronization, and lagging indices).

## Introduction

### Factors affecting economic growth rate

According to IHS Markit’s latest economic forecast for February 2020, the COVID-19 outbreak has dramatically reduced global demands and impacted supply chains, tourism, transportation, and international trade. The global economy has been greatly affected with its growth rate slowing down from the original 2.5% to 0.7%. Economic growth rate forecasts are of great importance to the future development of all countries in the world, and seeking out those factors affecting the changes in the economic environment is a topic of great concern by many global scholars and national governments [1].

In terms of economic growth-related literature, Kuzents [2] pointed out that the relationship between economic development and income distribution shows an inverted U-shape curve. Solow [3] pointed out in the economic growth theory that high savings lead to high outputs. Okun [4] showed that the economic growth falls by 2% for every 1% rise in the unemployment rate. Kuznets [5] also proposed the modern economic growth theory and pointed out that a country’s economic growth is determined by two major factors, and one is the country’s initial income level. Kuznets also proposed that the modern economic growth rate refers to a country’s GDP growth rate per capita, which is one type of economic development index. Krugman and Obstfeld [6] argued that the consumer price index can accurately reflect the price of a basket of goods. Barro [7] proposed the conditional convergence hypothesis, in which a country’s economy depends on its economic and environmental conditions (for example, labor growth rate, saving rate, population growth rate, government expenditure, etc.).

O'Higgins [8] found that a country’s unemployment rate is related to its overall economy, and the variables of the overall economy are NI, exchange rate, economic growth rate, CPI, etc. Naceur and Goaied [9] studied the relationship between interest margin and profit of a bank, showing a positive relationship between inflation rate and interest margin. Zaman and Mushtaq [10] explored the causal relationship among Pakistan’s GDP, input, output, and unemployment rate by a cointegration analysis method, and the results showed that an increase in GDP leads to an increase in employment population and a decline in the unemployment rate. Kim [11] and Karim [12] used the consumer price index and the wholesale price index to represent the price level.

Zhang et al. [13] noted that GDP growth has slowed markedly since 2008 based on data of China’s urban residents from 2005 to 2012, and that the urban unemployment rate also declined. Guo et al. [14] explored how an aging population affects economic growth and found that it has both positive and negative effects on economic growth. Chang et al. [15] analyzed the relationship between Americans’ actual GDP per capita and 6 indices of income inequality during the period from 1917 to 2012. Their results showed that the relationship between Americans’ actual average GDP and time and frequency inequality is of great significance to policy makers.

To study the effects of low fertility and aging population on economic growth, Teitelbaum [16] proposed the demographic transition theory that divides the demographic transition of countries into 3 stages: first stage—high birth rate and high death rate; second stage—high birth rate and low death rate; and third stage—low birth rate and low death rate.

Grey relational analysis (GRA) is a method which was proposed by Deng [17] mainly used to measure discrete sequences and to judge the relational grade between factors. GRA is widely used in many fields and successfully applied to solve a large number of practical problems in life and scientific research. For example, Krstic et al. [18] studied the effects (measured by the global competitiveness index [GCI]) of travel and tourism competitiveness on the economic competitiveness of sub-Saharan African (SSA) countries by regression, cluster, and GRA, and the results showed that travel and tourism competitiveness have significant effects on the competitiveness of the analyzed countries. Bao [19] evaluated the consistency between seven quantitative indices and those in the cleaner production reports of a group of three enterprises with the same nature through the improved analytic hierarchy process (AHP) model and GRA. The results were consistent with the cleaner production reports of the three enterprises, showing that the comprehensive method was feasible and could be a tool to evaluate internal cleaner production. Javed et. al. [20] introduces a new technique for the analysis of uncertain systems and uncertain processes in geothermics/earth sciences. The study concluded with valuable insights about the model and its application in engineering and natural sciences especially when the system contains uncertainty, which may arise either due to insufficient data or uncertain relationships among the parameters associated with the system or its processes. As the application of grey relational analysis is getting popular in variety of academic realms, Javanmardi [21] conducted a systematic review of grey systems theory-based methods and applications in sustainability studies.

Based on the literature review, grey relational analysis is a powerful mathematic tool to analyze the relationship among data sets. The method will be inapplicable if the time effect is unneglectable due to data nature. For example, the unemployment rate will not affect the economic rate simultaneously. It is usually deemed as lagging index. Therefore, the shift of raw data when the grey relational analysis is performed is required. Also, the difficulty of analysis is greatly increased and the computer program is needed to execute tedious calculation. In this study, the strategy of shifting raw data is called dynamic grey relational analysis. The innovative method will extend the grey rational analysis to even more application and contribute to the knowledge of grey systems theory (GST) to make GST more sound. This paper is organized as follows. Research method was used to introduce the mathematical tool adopted in this study. Empirical Results and Analysis showed the calculation process and presented numerical outcome. And the final section is conclusion to conclude the important findings of this research.

## Research method

The literature related to grey relation has employed static analysis in data studies, without considering leading or lagging time. A dynamic method (to compare the effects of sequential data movement) can be used to generate a time order, as well as the leading, synchronization, and lagging modes of a prosperity index. The present study realizes that more changes and combinations of original data could be generated by this movement method. In addition to that, there are various data preprocessing methods. Therefore, professional databases are needed to store larger data sizes. However, the current tools in the market do not meet the needs of this study. It is thus necessary to develop a customized system to process the tools needed. An innovative dynamic grey relational analysis is therefore used to explore the main factors affecting the economic growth rate.

### Data collection and software and hardware equipment

Based on the above data, birth rate (%), Taiwan’s total population (thousand people), unemployment rate (%), income per capita (USD), weighted average interest rate on deposits (%), Consumer Price Index, NI (million dollars), and economic growth rate are the factors discussed in this paper. Statistical data from 2006 to 2017 published on the statistical information network of Taiwan are used as the original data for numerical study. Microsoft asp.net c# and SQL2008 database are used as the software, and three personal computers are used as the hardware.

### Research process

A local GRA is adopted in this paper, and the explanations are as follows.

Step 1 Establish a reference sequence and a comparative sequence:

$$x_{0}(k)=x_{0}(1),x_{0}(2),\ldots x_{0}(n)$$ (1)

$$x_{i}(k)=x_{i}(1),x_{i}(2),....x_{i}(n)$$ (2)

where *k* = 1,2,3,⋯,*n*, *i* = 1,2,3,⋯,*m*. *x*_0_(*k*) is a reference sequence and *x*_*i*_(*k*) is a comparative sequence

Step 2 Standardize the original data:

$$(1)\;Initial\;value:\;x_{i}(k)=\frac{x_{i}(k)}{x_{i}(1)}$$ (3)

$$(2)\;\mathrm{Mean}\;\mathrm{value}:\;x_{i}(k)=\frac{x_{i}(k)}{\bar{x}}$$ (4)

$$(3)\;\mathrm{Percentage}:\;x_{i}(k)=\frac{x_{i}(k)}{\underset{k}{\max}x_{i}(k)}$$ (5)

$$(4)\;\mathrm{Multiple}:\;x_{i}(k)=\frac{x_{i}(k)}{\underset{k}{\min}x_{i}(k)}$$ (6)

$$(5)\;Maximum\;range:\;x_{i}(k)=\frac{x_{i}(k)-\underset{k}{\min}x_{i}(k)}{\underset{k}{\max}x_{i}(k)}$$ (7)

$$(6)\;Interval\;value:\;x_{i}(k)=\frac{x_{i}(k)-\underset{k}{\min}x_{i}(k)}{\underset{k}{\max}x_{i}(k)-\underset{k}{\min}x_{i}(k)}$$ (8)

Step 3 Obtain the grey relational coefficient:

$$The\;equation\;is:\;\gamma (x_{0}(k),x_{i}(k))=\frac{\Delta \min+\zeta \Delta \max}{\Delta_{oi}(k)+\zeta \Delta \max}$$

where *k* = 1,2,3,⋯,*n*, *i* = 1,2,3,⋯,*m*, *x*_0_ is the reference sequence, and *x*_*i*_ is a specific comparative sequence. *γ* is a grey relational coefficient

Δ_*oi*_ = ‖*x*_0_(*k*)−*x*_*i*_(*k*)‖: the absolute value of the *k*^th^ difference between *x*_0_ and *x*_*i*_.

$$\Delta_{\min.}=\overset{\min.\min.}{\underset{j\in i}{\forall }}\forall k\|x_{0}(k)-x_{j}(k)\|$$

$$\Delta_{\max.}=\overset{\max.\max.}{\underset{j\in i}{\forall }}\forall k\|x_{0}(k)-x_{j}(k)\|$$

*ζ*: distinguishing coefficient; *ζ*∈ [0,1]; in general, the distinguishing coefficient is 0.5 and can be adjusted as needed. The adjusted value will only change the relative value and will not affect the order of grey relational grade [18].

Step 4 Obtain the grey relational grade:

Obtain the mean value of the grey relational coefficient

$$The\;equation\;is:\;\gamma (x_{0},x_{i})=\frac{1}{n}\sum_{k=1}^{n}\gamma (x_{0}(k),x_{i}(k))$$

The closer the value of grey relational grade is to unity, the higher is the relational grade of the reference sequence; otherwise, it is lower.

Step 5 Obtain the grey relational order:

The grey relational grade indicates the relational grade between each sequence and the standard sequence. The order in which the relational grade are between all comparative sequences and the standard sequence is ranked by their values, and it is called the grey relational order.

When *γ*(*x*_0_,*x*_*i*_)>*γ*(*x*_0_,*x*_*j*_), it means that the relational grade between *x*_*i*_ and *x*_0_ is greater than that between *x*_*j*_ and *x*_0_; that is, *x*_*i*_ is more similar to *x*_0_.

Step 6 Computer system development:

In this system, Microsoft asp.net c# is used to develop the front-end web program, and the SQL2008 database is used in the back-end for access to the front-end web data. The system is divided into 3 stages for operation.

Phase 1 Import the data of Table 1 into SQL database and develop the first front-end web program by using Step 1. All comparative sequences are based on the data from 2009 to 2014. By randomly moving 0 to 3 grids, either to the left or to the right, six pieces of data can be continuously acquired to regenerate a comparative sequence. After movement, there are seven comparative sequences. Moreover, the original data from 2009 to 2014 are regularly used in the reference sequence. Therefore, a new set of original data is formed, and the original data generated by the above sequence are unrepeatable. In total, of 2,097,153 original data combinations were generated.
Phase 2 Develop 6 standardized front-end web programs by using Step 2, and each program transforms 2,097,153 combinations in the database into standard data.
Phase 3 Develop the 6^th^ front-end web program by using Step 3 to Step 5, and each program generates the final total relational grade for the standard data in the database.

**Table 1:** Original data from 2006 to 2017.

Year No. k Factor x sequence		2006	2007	2008	2009	2010	2011	2012	2013	2014	2015	2016	2017
		K1	K2	K3	K4	K5	K6	K7	K8	K9	K10	K11	K12
Reference sequence (x0)	Economic growth Rate	5.62	6.52	0.7	-1.57	10.63	3.8	2.06	2.2	4.02	0.81	1.41	2.86
Comparative sequence (x1)	Birth Rate (%)	8.96	8.92	8.64	8.29	7.21	8.48	9.86	8.53	8.99	9.1	8.86	8.23
Comparative sequence (x2)	Taiwan’s total population (thousand people)	22876.53	22958.36	23037.03	23119.77	23162.12	23224.91	23315.82	23373.52	23433.75	23492.07	23539.82	23571.23
Comparative sequence (x3)	Unemployment Rate (%)	3.91	3.91	4.14	5.85	5.21	4.39	4.24	4.18	3.96	3.78	3.92	3.76
Comparative sequence (x4)	Income per capita (USD)	14974	15401	15388	14398	16650	17982	18125	18872	19724	19571	19720	21094
Comparative sequence (x5)	Weighted average interest rate on deposits (%)	1.4	1.53	1.71	0.85	0.61	0.75	0.82	0.8	0.78	0.77	0.63	0.56
Comparative sequence (x6)	Consumer Price Index (CPI)	0.6	1.8	3.52	-0.87	0.97	1.42	1.93	0.79	1.2	-0.3	1.39	0.62
Comparative sequence (x7)	National Income (million dollars)	11117367	11590959	11161869	10985329	12194428	12290671	12493108	13115430	14018941	14652714	14992247	15120728

### Empirical results and analysis

According to the final execution results of system development in Phase 3, the closer the characteristic index of grey relational grade is to unity, the higher is the relational grade between the comparative sequence and the reference sequence; otherwise, it is lower. However, there are 2,097,153 groups of relational grade in each standardization. In this study, the relational grade of all groups are added up to obtain the group with the maximum total value of relational grade for subsequent analysis.

### The ranking of grey relational grade

The maximum total values of six standardized dynamic and static relational grades are taken from the database, and all the relational orders are organized and ranked from the largest to the smallest, as shown in Table 2. The seven factors of all relational orders in Table 2 are ranked from the largest to the smallest and divided into (relation ranking 1, 2, 3, 4, 5, 6, 7) 7 groups, as shown in Table 3. The frequencies of appearance of all factors are added up by using the 7 ranking groups in Table 3. The results are shown in Tables 4 and 5.

**Table 2:** Relational grades and relational orders of all standardizations.

Initial value (relational order_ relational grade)	
Static grey relation	[x6_0.7866]>[x3_0.6266]>[x5_0.6146]>[x2_0.6087]>[x1_0.6051]>[x7_0.5989]>[x4_0.5925]
Dynamic grey relation	[x5_0.6859]>[x3_0.6668]>[x1_0.6568]>[x2_0.6498]>[x7_0.6485]>[x4_0.6465]>[x6_0.6321]
Mean value (relational order_ relational grade)	
Static grey relation	[x4_0.7092]>[x7_0.7070]>[x2_0.6941]>[x1_0.6862]>[x3_0.6834]>[x5_0.6774]>[x6_0.6517]
Dynamic grey relation	[x7_0.7976]>[x2_0.7947]>[x4_0.7945]>[x3_0.7929]>[x1_0.7919]>[x5_0.7866]>[x6_0.6581]
Percentage (relational order_ relational grade)	
Static grey relation	[x6_0.6206]>[x3_0.5908]>[x2_0.5287]>[x7_0.5260]>[x4_0.5179]>[x1_0.5069]>[x5_0.4942]
Dynamic grey relation	[x5_0.7623]>[x6_0.6277]>[x3_0.6142]>[x1_0.5682]>[x7_0.5390]>[x4_0.5383]>[x2_0.5357]
Multiple (relational order_ relational grade)	
Static grey relation	[x6_0.7893]>[x2_0.6127]>[x7_0.6029]>[x4_0.5965]>[x1_0.5937]>[x3_0.5908]>[x5_0.5823]
Dynamic grey relation	[x6_0.8111]>[x2_0.6459]>[x1_0.6429]>[x7_0.6425]>[x4_0.6418]>[x3_0.6401]>[x5_0.5868]
Maximum range (relational order_ relational grade)	
Static grey relation	[x4_0.7160]>[x7_0.6720]>[x1_0.6685]>[x5_0.6569]>[x2_0.6090]>[x6_0.5982]>[x3_0.5698]
Dynamic grey relation	[x5_0.7361]>[x4_0.7295]>[x3_0.7197]>[x1_0.7146]>[x7_0.6886]>[x6_0.6388]>[x2_0.6262]
Interval value (relational order_ relational grade)	
Static grey relation	[x7_0.6982]>[x6_0.6428]>[x2_0.6296]>[x4_0.6109]>[x1_0.6090]>[x3_0.6075]>[x5_0.5139]
Dynamic grey relation	[x3_0.7043]>[x6_0.6866]>[x5_0.6798]>[x2_0.6498]>[x4_0.6433]>[x7_0.6136]>[x1_0.6090]

**Table 3:** Ranking of 7 factors.

	Initial value	Mean value	Percentage	Multiple	Maximum range	Interval value
Ranking 1 of 6 standardizations						
Static grey relation	Consumer Price Index	Income per capita (USD)	Consumer Price Index	Consumer Price Index	Income per capita (USD)	National Income (million dollars)
Dynamic grey relation	Weighted average interest rate on deposits(%)	National Income (million dollars)	Weighted average interest rate on deposits(%)	Consumer Price Index	Weighted average interest rate on deposits(%)	Unemployment Rate(%)
Ranking 2 of 6 standardizations						
Static grey relation	Unemployment Rate(%)	National Income (million dollars)	Unemployment Rate(%)	Taiwan’s total population (thousand people)	National Income (million dollars)	Consumer Price Index
Dynamic grey relation	Unemployment Rate(%)	Taiwan’s total population (thousand people)	Consumer Price Index	Taiwan’s total population (thousand people)	Income per capita (USD)	Consumer Price Index
Ranking 3 of 6 standardizations						
Static grey relation	Weighted average interest rate on deposits(%)	Taiwan’s total population (thousand people)	Taiwan’s total population (thousand people)	National Income (million dollars)	Birth Rate(%)	Taiwan’s total population (thousand people)
Dynamic grey relation	Birth Rate(%)	Income per capita (USD)	Unemployment Rate(%)	Birth Rate(%)	Unemployment Rate(%)	Weighted average interest rate on deposits(%)
Ranking 4 of 6 standardizations						
Static grey relation	Taiwan’s total population (thousand people)	Birth Rate(%)	National Income (million dollars)	Income per capita (USD)	Weighted average interest rate on deposits(%)	Income per capita (USD)
Dynamic grey relation	Taiwan’s total population (thousand people)	Unemployment Rate(%)	Birth Rate(%)	National Income (million dollars)	Birth Rate(%)	Taiwan’s total population (thousand people)
Ranking 5 of 6 standardizations						
Static grey relation	Birth Rate(%)	Unemployment Rate(%)	Income per capita (USD)	Birth Rate(%)	Taiwan’s total population (thousand people)	Birth Rate(%)
Dynamic grey relation	National Income (million dollars)	Birth Rate(%)	National Income (million dollars)	Income per capita (USD)	National Income (million dollars)	Income per capita (USD)
Ranking 6 of 6 standardizations						
Static grey relation	National Income (million dollars)	Weighted average interest rate on deposits(%)	Birth Rate(%)	Unemployment Rate(%)	Consumer Price Index	Unemployment Rate(%)
Dynamic grey relation	Income per capita (USD)	Weighted average interest rate on deposits(%)	Income per capita (USD)	Unemployment Rate(%)	Consumer Price Index	National Income (million dollars)
Ranking 7 of 6 standardizations						
Static grey relation	Income per capita (USD)	Consumer Price Index	Weighted average interest rate on deposits(%)	Weighted average interest rate on deposits(%)	Unemployment Rate(%)	Weighted average interest rate on deposits(%)
Dynamic grey relation	Consumer Price Index	Consumer Price Index	Taiwan’s total population (thousand people)	Weighted average interest rate on deposits(%)	Taiwan’s total population (thousand people)	Birth Rate(%)

**Table 4:** Frequencies of all factors (static grey relation).

Ranking Factor	Ranking 1	Ranking 2	Ranking 3	Ranking 4	Ranking 5	Ranking 6	Ranking 7
Birth Rate(%)	0	0	1	1	3	1	0
Taiwan’s total population (thousand people)	0	1	3	1	1	0	0
Unemployment Rate(%)	0	2	0	0	1	2	1
Income per capita (USD)	2	0	0	2	1	0	1
Weighted average interest rate on deposits(%)	0	0	1	1	0	1	3
Consumer Price Index	3	1	0	0	0	1	1
National Income (million dollars)	1	2	1	1	0	1	0

**Table 5:** Frequencies of all factors (dynamic grey relation).

Ranking Factor	Ranking 1	Ranking 2	Ranking 3	Ranking 4	Ranking 5	Ranking 6	Ranking 7
Birth Rate (%)	0	0	2	2	1	0	1
Taiwan’s total population (thousand people)	0	2	0	2	0	0	2
Unemployment Rate (%)	1	1	2	1	0	1	0
Income per capita (USD)	0	1	1	0	2	2	0
Weighted average interest rate on deposits (%)	3	0	1	0	0	1	1
Consumer Price Index	1	2	0	0	0	1	2
National Income (million dollars)	1	0	0	1	3	1	0

The factors with the highest frequencies in all rankings are selected from Table 4. The relational orders are re-ranked from large to small as follows: CPI > (NI (million dollars), unemployment rate (%) > Taiwan’s total population (thousand people) > income per capita (USD) > birth rate > unemployment rate (%) > weighted average interest rate on deposits (%), unemployment rate (%). The unemployment rate appeared twice in succession, respectively ranking 2 and 6. Thus, the new ranking is simplified to: CPI > (NI (million dollars), unemployment rate (%) > Taiwan’s total population (thousand people) > income per capita (USD) > birth rate > weighted average interest rate on deposits (%).

The factors with the highest total frequencies in all rankings are selected from Table 5. The relational orders are re-ranked from large to small as follows: weighted average interest rate on deposits (%) > (Taiwan’s total population (thousand people), CPI) > (birth rate, unemployment rate (%)) > (birth rate, Taiwan’s total population (thousand people)) > NI (million dollars) > income per capita (USD) > (Taiwan’s total population (thousand people), CPI). Taiwan’s total population appears three times in succession, respectively ranking 2, 4, and 7, and birth rate appears twice in succession, respectively ranking 3 and 4, and so the new relational order is simplified to: weighted average interest rate on deposits (%) > (Taiwan’s total population (thousand people), CPI) > (birth rate, unemployment rate (%)) > Taiwan’s total population (thousand people) > NI (million dollars) > income per capita (USD) > CPI, Taiwan’s total population (thousand people).

### Distribution of prosperity indices of all factors

The static and dynamic grey relations are integrated and compared, as shown in Table 6. The relational grades of initial value *x*6 (CPI) and interval value *x*7 (NI (million dollars)) decline, and the relational grade of interval value *x*1 (birth rate) remains unchanged, whereas other relational grades increase. The data from 2009 to 2014 are synchronous—namely, the synchronization indices—and Table 1 is moved up three grids to the left or right according to the dynamic grey relational movement and divided into three zones, including the lagging index from 2006 to 2008 and the leading index from 2015 to 2017, as organized in Table 7. After that, the data are moved to a maximum of three grids, with each grid representing one year. The indices are subdivided as shown in Table 8. Finally, the original data are retrieved from the database according to the grey relational grade, to create Table 9.

**Table 6:** Rise and decline of relational grade.

Initial value							
	x1	x2	x3	x4	x5	x6	x7
Static grey relation	0.6051	0.6087	0.6266	0.5925	0.6146	0.7866	0.5989
Dynamic grey relation	0.6568	0.6498	0.6668	0.6465	0.6859	0.6321	0.6485
Rise / decline of relational grade	Rise	Rise	Rise	Rise	Rise	降	Rise
Mean value							
	x1	x2	x3	x4	x5	x6	x7
Static grey relation	0.6862	0.6941	0.6834	0.7092	0.6774	0.6517	0.707
Dynamic grey relation	0.7919	0.7947	0.7929	0.7945	0.7866	0.6581	0.7976
Rise / decline of relational grade	Rise	Rise	Rise	Rise	Rise	Rise	Rise
Percentage							
	x1	x2	x3	x4	x5	x6	x7
Static grey relation	0.5069	0.5287	0.5908	0.5179	0.4942	0.6206	0.526
Dynamic grey relation	0.5682	0.5357	0.6142	0.5383	0.7623	0.6277	0.539
Rise / decline of relational grade	Rise	Rise	Rise	Rise	Rise	Rise	Rise
Multiple							
	x1	x2	x3	x4	x5	x6	x7
Static grey relation	0.5937	0.6127	0.5908	0.5965	0.5823	0.7893	0.6029
Dynamic grey relation	0.6429	0.6459	0.6401	0.6418	0.5868	0.8111	0.6425
Rise / decline of relational grade	Rise	Rise	Rise	Rise	Rise	Rise	Rise
Maximum range							
	x1	x2	x3	x4	x5	x6	x7
Static grey relation	0.6685	0.609	0.5698	0.716	0.6569	0.5982	0.672
Dynamic grey relation	0.7146	0.6262	0.7197	0.7295	0.7361	0.6388	0.6886
Rise / decline of relational grade	Rise	Rise	Rise	Rise	Rise	Rise	Rise
Interval value							
	x1	x2	x3	x4	x5	x6	x7
Static grey relation	0.609	0.6296	0.6075	0.6109	0.5139	0.6428	0.6982
Dynamic grey relation	0.609	0.6498	0.7043	0.6433	0.6798	0.6866	0.6136
Rise / decline of relational grade	constant	Rise	Rise	Rise	Rise	Rise	decline

**Table 7:** Comparison of prosperity indices (original data from 2006 to 2017).

Year Index No k Factor x sequence		2006	2007	2008	2009	2010	2011	2012	2013	2014	2015	2016	2017
		Lagging index			Synchronization index						Leading index		
		K1	K2	K3	K4	K5	K6	K7	K8	K9	K10	K11	K12
Reference sequence (x0)	Economic growth Rate	5.62	6.52	0.7	-1.57	10.63	3.8	2.06	2.2	4.02	0.81	1.41	2.86
Comparative sequence (x1)	Birth Rate (%)	8.96	8.92	8.64	8.29	7.21	8.48	9.86	8.53	8.99	9.1	8.86	8.23
Comparative sequence (x2)	Taiwan’s total population (thousand people)	22876.53	22958.36	23037.03	23119.77	23162.12	23224.91	23315.82	23373.52	23433.75	23492.07	23539.82	23571.23
Comparative sequence (x3)	Unemployment Rate (%)	3.91	3.91	4.14	5.85	5.21	4.39	4.24	4.18	3.96	3.78	3.92	3.76
Comparative sequence (x4)	Income per capita (USD)	14974	15401	15388	14398	16650	17982	18125	18872	19724	19571	19720	21094
Comparative sequence (x5)	Weighted average interest rate on deposits (%)	1.4	1.53	1.71	0.85	0.61	0.75	0.82	0.8	0.78	0.77	0.63	0.56
Comparative sequence (x6)	Consumer Price Index (CPI)	0.6	1.8	3.52	-0.87	0.97	1.42	1.93	0.79	1.2	-0.3	1.39	0.62
Comparative sequence (x7)	National Income (million dollars)	11117367	11590959	11161869	10985329	12194428	12290671	12493108	13115430	14018941	14652714	14992247	15120728

**Table 8:** Subdivision and comparison of prosperity indices.

(x sequence, k sequence) n = 1.2.3.	Index
(xn,k1)~ (xn,k6)	Lagging index (lagging for 3 years)
(xn,k2)~ (xn,k 7)	Lagging index (lagging for 2 years)
(xn,k3)~ (xn,k 8)	Lagging index (lagging for 1 years)
(xn,k4)~ (xn,k 9)	Synchronization index
(xn,k7)~ (xn,k 12)	Leading index (leading for 3 years)
(xn,k6)~ (xn,k 11)	Leading index (leading for 2 years)
(xn,k5)~ (xn,k 10)	Leading index (leading for 1 years)

**Table 9:** Subdivision and comparison of prosperity indices.

Factor Data	Economic growth Rate (%)	Birth Rate(%)	Taiwan’s total population (thousand people)	Unemployment Rate(%)	Income per capita (USD)	Weighted average interest rate on deposits (%)	Consumer Price Index (CPI)	National Income (million dollars)
	Initial value							
Original data	(x0,k4~k9)	(x1,k4~k9)	(x2,k4~k9)	(x3,k4~k9)	(x4,k4~k9)	(x5,k4~k9)	(x6,k4~k9)	(x7,k4~k9)
Original data (movement)	(x0,k4~k9)	(x1,k7~k12)	(x2,k7~k12)	(x3,k4~k9)	(x4,k1~k6)	(x5,k3~k8)	(x6,k1~k6)	(x7,k2~k7)
	Mean value							
Original data	(x0,k4~k9)	(x1,k4~k9)	(x2,k4~k9)	(x3,k4~k9)	(x4,k4~k9)	(x5,k4~k9)	(x6,k4~k9)	(x7,k4~k9)
Original data (movement)	(x0,k4~k9)	(x1,k6~k11)	(x2,k2~k7)	(x3,k7~k12)	(x4,k5~k11)	(x5,k1~k6)	(x6,k3~k8)	(x7,k1~k6)
	Percentage							
Original data	(x0,k4~k9)	(x1,k4~k9)	(x2,k4~k9)	(x3,k4~k9)	(x4,k4~k9)	(x5,k4~k9)	(x6,k4~k9)	(x7,k4~k9)
Original data (movement)	(x0,k4~k9)	(x1,k6~k11)	(x2,k6~ k11)	(x3,k3~k8)	(x4,k1~k6)	(x5,k2~k7)	(x6,k3~k8)	(x7,k1~k6)
	Multiple							
Original data	(x0,k4~k9)	(x1,k4~k9)	(x2,k4~k9)	(x3,k4~k9)	(x4,k4~k9)	(x5,k4~k9)	(x6,k4~k9)	(x7,k4~k9)
Original data (movement)	(x0,k4~k9)	(x1,k6~k11)	(x2,k7~k12)	(x3,k7~k12)	(x4,k6~k11)	(x5,k2~k7)	(x6,k4~k9)	(x7,k1~k6)
	Maximum range							
Original data	(x0,k4~k9)	(x1,k4~k9)	(x2,k4~k9)	(x3,k4~k9)	(x4,k4~k9)	(x5,k4~k9)	(x6,k4~k9)	(x7,k4~k9)
Original data (movement)	(x0,k4~k9)	(x1,k5~k10)	(x2,k1~k6)	(x3,k1~k6)	(x4,k4~k9)	(x5,k5~k10)	(x6,k3~k8)	(x7,k7~k12)
	Interval value							
Original data	(x0,k4~k9)	(x1,k4~k9)	(x2,k4~k9)	(x3,k4~k9)	(x4,k4~k9)	(x5,k4~k9)	(x6,k4~k9)	(x7,k4~k9)
Original data (movement)	(x0,k4~k9)	(x1,k4~k9)	(x2,k5~k10)	(x3,k7~k12)	(x4,k5~k10)	(x5,k2~k7)	(x6,k6~k11)	(x7,k6~k11)

The prosperity indices of all factors of six data preprocessing methods are organized as shown in Table 10, by using Table 9 for the coordinate codes of original data and Table 7. As seen, except for the interval value of *x*1 (birth rate) being synchronous, the other standardizations are more relevant if they are more into the lead. Aside from the mean value and the maximum range being in the lag, other values of *x*2 (Taiwan’s total population (thousand people)) are more relevant if they are more in the lead. Aside from the maximum range being in the lead, other values of *x*5 (weighted average interest rate on deposits (%)) are more relevant if they are more in the lag. Aside from the multiple being synchronous and the interval value being in the lead, other values of x6 (CPI) are more relevant if they are more in the lag. Aside from the maximum range and the interval value being in the lead, other values of *x*7 (NI (million dollars)) are more relevant if they are more in the lag. The analysis on the prosperity indices of other factors is shown in Table 10.

**Table 10:** Prosperity index comparison.

Initial value							
	x1	x2	x3	x4	x5	x6	x7
Static grey relation	Synchronization	Synchronization	Synchronization	Synchronization	Synchronization	Synchronization	Synchronization
Dynamic grey relation	Leading 3	Leading 3	Synchronization	Lagging 3	Lagging 1	Lagging 3	Lagging 2
Mean value							
	x1	x2	x3	x4	x5	x6	x7
Static grey relation	Synchronization	Synchronization	Synchronization	Synchronization	Synchronization	Synchronization	Synchronization
Dynamic grey relation	Leading 2	Lagging 2	Leading 3	Leading 1	Lagging 3	Lagging 1	Lagging 3
Percentage							
	x1	x2	x3	x4	x5	x6	x7
Static grey relation	Synchronization	Synchronization	Synchronization	Synchronization	Synchronization	Synchronization	Synchronization
Dynamic grey relation	Leading 2	Leading 2	Lagging 1	Lagging 3	Lagging 2	Lagging 1	Lagging 3
Multiple							
	x1	x2	x3	x4	x5	x6	x7
Static grey relation	Synchronization	Synchronization	Synchronization	Synchronization	Synchronization	Synchronization	Synchronization
Dynamic grey relation	Leading 2	Leading 3	Leading 3	Leading 2	Lagging 2	Synchronization	Lagging 3
Maximum range							
	x1	x2	x3	x4	x5	x6	x7
Static grey relation	Synchronization	Synchronization	Synchronization	Synchronization	Synchronization	Synchronization	Synchronization
Dynamic grey relation	Leading 1	Lagging 3	Lagging 3	Synchronization	Leading 1	Lagging 1	Leading 3
Interval value							
	x1	x2	x3	x4	x5	x6	x7
Static grey relation	Synchronization	Synchronization	Synchronization	Synchronization	Synchronization	Synchronization	Synchronization
Dynamic grey relation	Synchronization	Leading 1	Leading 3	Leading 1	Lagging 2	Leading 2	Leading 2

The prosperity indices of the static grey relation in Table 10 are changed to those of the dynamic grey relation, and the data movement directions are organized into Table 11. As seen, the standardized prosperity indices closer to the left are more in the lead except that the prosperity indices of the interval value of birth rate are synchronous. The standardized prosperity indices closer to the right are more in the lead, except that the prosperity indices of the maximum range of weighted average interest rate on deposits (%) and NI (million dollars) are closer to the left. The standardized prosperity indices closer to the right are more in the lead, except that the prosperity indices of the multiple of CPI were synchronous and the prosperity indices of the interval value are closer to the left. Table 11 shows the analysis on the prosperity indices of other factors.

**Table 11:** Directions of prosperity indices of all factors—changes from static data to dynamic data.

Standardization Factor	Initial value	Mean value	Percentage	Multiple	Maximum range	Interval value
Birth Rate (%)	To the left	To the left	To the left	To the left	To the right	Synchronization
Taiwan’s total population (thousand people)	To the left	To the right	To the left	To the left	To the right	To the left
Unemployment Rate (%)	Synchronization	To the left	To the right	To the left	To the right	To the left
Income per capita (USD)	To the right	To the right	To the right	To the left	Synchronization	To the left
Weighted average interest rate on deposits (%)	To the right	To the right	To the right	To the right	To the left	To the right
Consumer Price Index	To the right	To the right	To the right	Synchronization	To the right	To the left
National Income (million dollars)	To the right	To the right	To the right	To the right	To the left	To the right

## Conclusions

In traditional studies on the economic growth rate by grey relation, the original data were collected and preprocessed by a selected method. The data used are static to finally generate a set of relational order, but different data preprocessing methods result in different rankings. Under inconsistent conditions, in this study, the grey relation was divided into dynamic and static analyses by using an innovative method, and the characteristic of moving original data dynamically was used, in order to finally integrate various data preprocessing methods and generate a new set of rankings. The time order of the original data produced by various factors was formed in a dynamic manner, to generate new data corresponding to the prosperity indices (leading, synchronization, and lagging). The final results can be used to solve the inconsistency arising from the use of the different data pre-processing methods.

There is no past literature talking about dynamic grey relation. It was thus found in this study that the ranking of static grey relation changes after dynamic grey relation, and that the relational grade increased or decreased. If the grade of decline in the relational grade can be found—i.e., deleting the affecting factors—then the accuracy of the study on affecting factors can be improved. The integrated achievements of this study are noted below.

1. In the process of moving the original data from dynamic grey relation, the original data should not be less than that of the static grey relation, and the number of grids of original data movement should be greater than the number of grids of original data movement of this study.
2. In this study, 6 data preprocessing methods were used for dynamic grey relational analysis, and the results showed that, except for the relational grades of initial value x6 (consumer price index) and interval value *x*7 (NI (million dollars)) in the static grey relation declining after dynamic grey relation, the relational grades of other factors were higher than those in the original static grey relation. In addition, any factor originally ranking first in the static grey relation might become last after dynamic grey relational analysis.
3. The prosperity indices were used to identify which factors are the leading, synchronization, and lagging indices affecting economic growth, so as to verify whether the static state (synchronization) has a leading or lagging relation.
